# Chest Pain as a Symptom of Early-Onset Metastatic Prostate Cancer: Exploring the Role of Screening

**DOI:** 10.7759/cureus.78143

**Published:** 2025-01-28

**Authors:** Fizza Mohsin, Brent Boodhai, Aarti Maharaj, Nissim Levy, Muhammad Hashim Khan, Fatima Sajid, Shaurya Sharma

**Affiliations:** 1 Medicine, Maimonides Medical Center, New York, USA; 2 Pulmonary and Critical Care Medicine, University of Florida College of Medicine - Jacksonville, Jacksonville, USA; 3 Internal Medicine, University of Florida College of Medicine - Jacksonville, Jacksonville, USA; 4 Internal Medicine, Maimonides Medical Center, New York, USA

**Keywords:** brca gene mutation, chestpain, early onset, prostate cancer, screening

## Abstract

Prostate cancer is one of the most prevalent malignancies affecting males, primarily occurring in older men. However, it has been observed that there is a growing trend of new cases among younger individuals. We present the case of a 35-year-old male who had experienced persistent, severe right-sided chest pain for three months, unrelieved by analgesics. An inpatient workup revealed elevated alkaline phosphatase, prompting a more thorough evaluation that included measuring prostate-specific antigen (PSA) levels. His CT scan revealed sclerotic lesions in the ribs concerning metastatic disease. A biopsy of the enlarged retroperitoneal lymph nodes confirmed the diagnosis of metastatic prostate adenocarcinoma. Due to the patient's age and strong family history, genetic testing was conducted, revealing a BRCA2 mutation. Chest pain in a young patient can be misleading, and his symptoms did not initially suggest prostate cancer as a primary differential diagnosis. However, the persistence of symptoms, imaging results, and a strong family history of prostate cancer directed the physicians toward the correct diagnosis. Additionally, BRCA2 mutations are linked to a more aggressive form of the disease, and a timely diagnosis can lead to better outcomes. However, there is limited data regarding routine screening in high-risk patients such as African Americans and those with genetic predisposition. Therefore, healthcare providers should collaborate with their patients to develop personalized screening plans.

## Introduction

Prostate cancer is the most frequently diagnosed malignancy in men and ranks as the fifth leading cause of cancer-related death among men globally. From 2017 to 2021, the incidence rate was 116.5 per 100,000 men annually [1]. While it predominantly affects older men, the occurrence of early-onset prostate cancer (EOPC) has doubled in the past 20 years, highlighting it as an emerging public health issue. EOPC cases often exhibit higher cancer-related mortality rates and are linked to genetic mutations and more advanced disease stages. This case report aims to raise awareness of the rising incidence of EOPC and its genetic predisposition. It was previously presented as a meeting abstract at the Coverage from the American College of Chest Physicians (CHEST) Annual Scientific Meeting on October 18, 2022.

## Case presentation

A 35-year-old African American man presented with severe right-sided chest pain lasting three days. The pain was sharp in nature and worsened with spinal flexion, extension, and chest palpation, significantly restricting the patient's ability to perform activities of daily living (ADLs). Three months earlier, he had experienced a similar sharp, persistent anterior left-sided chest pain, which later spread to the right chest, lower back, and shoulders. He had previously visited outpatient medical services three times, during which serial electrocardiograms (EKGs), troponins, and a stress test yielded negative results. A trial of H2-receptor blockers was initiated to rule out gastroesophageal reflux disease (GERD). Additionally, an echocardiogram revealed no significant findings. As a cable installation technician who occasionally engaged in heavy lifting, his pain was initially attributed to his job's physical demands and was considered musculoskeletal in nature. However, analgesia provided no relief. His medical history included sickle cell trait, and he had a family history of prostate cancer, with his father, paternal grandfather, and paternal uncle having been diagnosed. Vital signs were within normal ranges. During further discussion, the patient mentioned experiencing an occasional weak urinary stream and frequent nighttime awakenings to urinate, which he mainly attributed to dehydration and late-night coffee consumption. Physical examination revealed diffuse tenderness upon palpation of the 5th-6th intercostal spaces bilaterally, as well as bilateral scapular tenderness. The chest was clear to auscultation, with normal S1 and S2 heart sounds and no murmurs. Positive bowel sounds and intact reflexes were noted, with no signs of leg swelling or edema. Laboratory results, including a prostate-specific antigen (PSA) level of 2945.28 ng/ml, are summarized in Table 1.

**Table 1:** Laboratory results

Parameter	Result	Reference ranges
Hemoglobin	11.2 g/dl	14-18 g/dl
Mean corpuscular volume	77.5 fl	80-94 fl
White blood cells	7.5 k/ul	4.8-10.8 k/uL
Platelets	197 k/ul	150-450 k/ul
Blood urea nitrogen	11.39 mg/dl	7-21 mg/dl
Creatinine	1 mg/dl	0/5-1.3 mg/dl
Total bilirubin	0.7 mg/dl	0.2-1.4 g/dl
Direct bilirubin	0.3 mg/dl	0-0.2 mg/dl
Aspartate aminotransferase	17 IU/L	36-112 IU/L
Alanine transaminase	10 IU/L	6-47 IU/L
Alkaline phosphatase	937 IU/L	36-112 IU/L
Carcinoembryonic antigen	20.2 ng/ml	0.3-2.5ng/ml
Prostate-specific antigen	2945 ng/ml	0.01-3.80 ng/ml
Cancer antigen 125	11.6 U/ml	0.0-35.0 U/ml
Alpha-fetoprotein	3.2 ng/ml	<83 ng/ml
Human chorionic gonadotropin	1 mIU/ml	<1 mIU/ml

**Figure 1:**
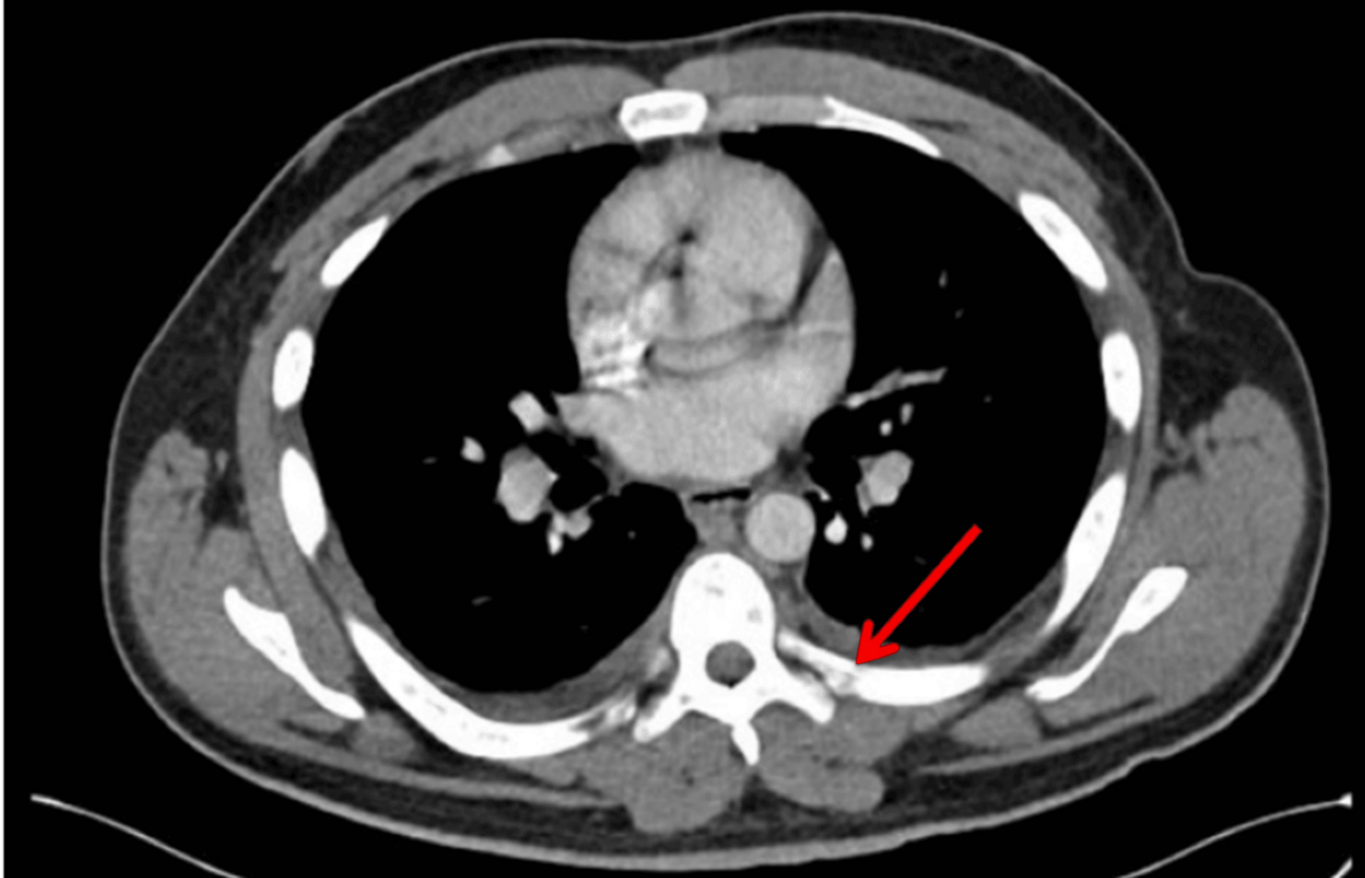
CT scan showing a sclerotic lesion with cortical breakdown in the left posterior sixth rib (red arrow)

**Figure 2:**
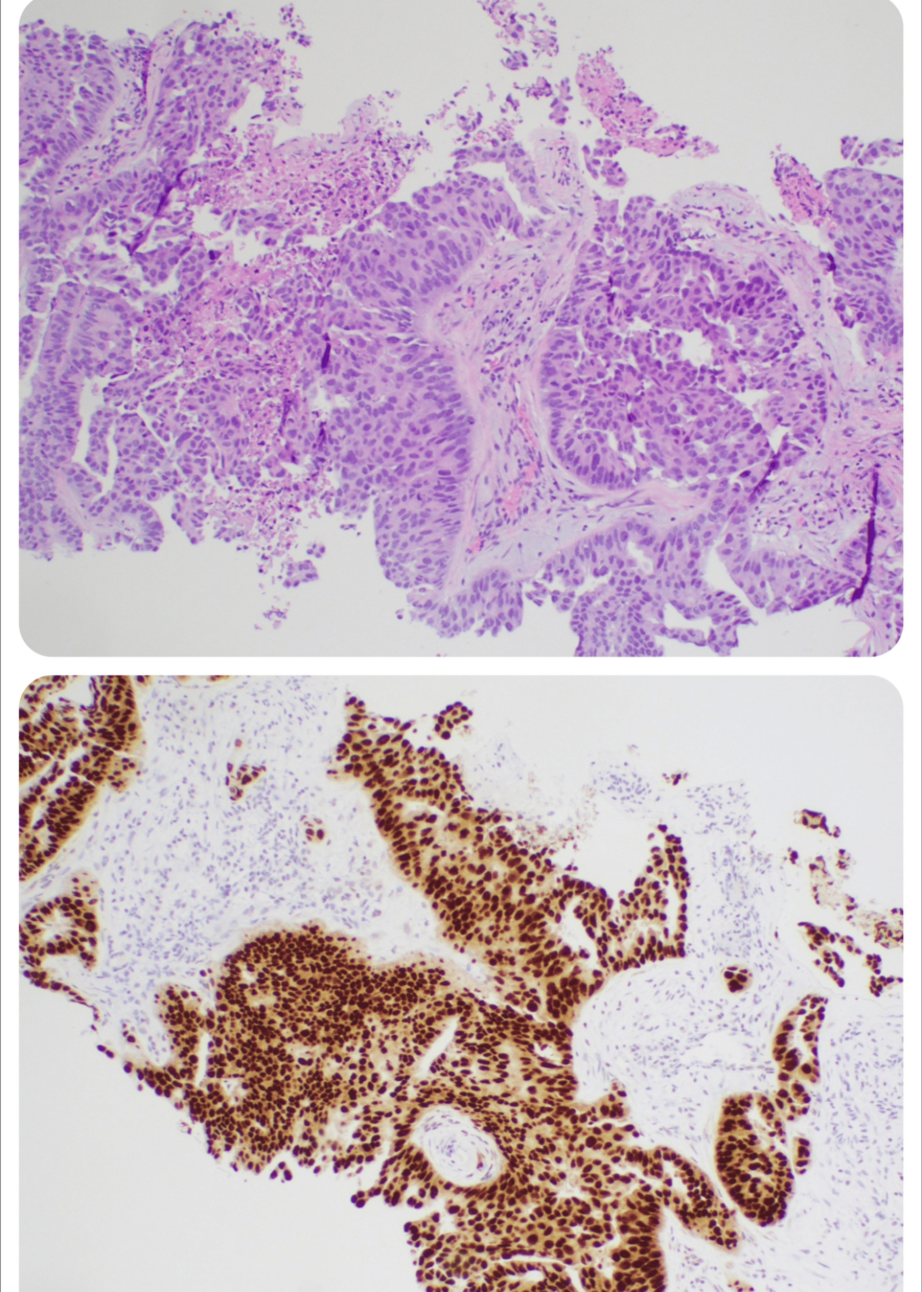
Prostate biopsy showing adenocarcinoma (optical microscopy with hematoxylin and eosin staining (top) and immunohistochemistry (IHC) staining (bottom), viewed at 100× magnification)

The CT scan of the chest revealed numerous sclerotic lesions throughout the skeleton, including the scapula, lumbar and thoracic spine, pelvis, and right femur. Cortical breakdown was observed in the left posterior sixth rib, raising suspicion of metastatic disease (Figure 1). A CT scan of the abdomen and pelvis revealed an irregularly shaped prostate, hypodense areas, and retroperitoneal lymphadenopathy, which was confirmed by a transrectal ultrasound (TRUS) biopsy as metastatic prostate adenocarcinoma. The prostate biopsy revealed prostate adenocarcinoma in all 12 samples collected. The Gleason scores ranged from 4/5 + 4/5 = 8/10 to 5/5 + 4/5 = 9/10. (Figure 2). Genetic analysis revealed a positive BRCA2 mutation, detected in both the germline DNA analysis and the somatic mutation profile of the tumor. Given the advanced stage of the disease and the presence of distant metastasis, systemic therapy was chosen as the primary treatment approach. The extensive local involvement observed in the metastatic disease led to the use of well-established systemic anti-androgen monotherapy with bicalutamide 150 mg once daily. The patient's mobility and pain improved significantly after the start of treatment. Progression and recovery have been closely monitored through clinical assessments, liver function tests, imaging studies, and regular follow-up. PSA levels were also carefully tracked.

## Discussion

Prostate cancer ranks among the top two most prevalent non-cutaneous cancers in men. Its presentation may include atypical symptoms that can lead to an incorrect initial diagnosis [2]. Prostate cancer is characterized by both heritable and acquired mutations in the genome. Lange et al.'s findings demonstrated the more significant role of genetic factors in the occurrence of EOPC compared to later-onset cases [3]. The significance of EOPC may have been previously overlooked. Yet, with increasing prevalence and more extensive screening practices, approximately 10% of diagnosed cases now occur in men aged 55 years or younger. This shift in prostate cancer epidemiology toward younger age groups can be predominantly attributed to the adoption of PSA-based screening methods [4]. EOPC often exhibits a strong inherited predisposition, notably in cases where a BRCA2 mutation is identified [5]. Patients with BRCA2 mutations are at a heightened risk of developing more aggressive forms of the disease. The aggressive nature of EOPC significantly influences treatment decisions compared to late-onset prostate cancer (LOPC). EOPC is often diagnosed with a higher Gleason score or at a more advanced stage, requiring more intensive and aggressive treatment approaches [6]. Bones are a preferential site of metastasis in prostate cancer, with synchronous involvement most commonly occurring in the lungs and liver. Given the higher likelihood of early metastasis in EOPC, including to the bones and lymph nodes, treatments such as bone-targeted therapies (e.g., radium-223) and chemotherapy are often necessary to manage widespread disease [7]. Most professional societies advocate for high-risk patients to engage in shared decision-making with healthcare providers to devise personalized screening plans. Clinicians should be familiar with DNA repair pathway alterations associated with prostate cancer. For instance, prostate cancer with mutations in homologous recombination repair genes such as BRCA1 and BRCA2 shows increased sensitivity to poly (ADP-ribose) polymerase (PARP) inhibitors and may benefit from platinum chemotherapy. Conversely, PD-1 inhibitors can target mutations in genes like MLH1, MSH2, MSH6, and PMS2, linked to microsatellite instability and mismatch repair deficiency [8,9]. With the increasing utilization of genetic testing in prostate cancer patients, precision therapy options are becoming more accessible [10]. However, patients should receive counseling both before and after testing, as results may sometimes yield inconclusive variations. Identifying mutations can also aid in assessing patients for secondary malignancies and identifying at-risk relatives for various cancers [11].

Differentiating between benign causes of chest pain in young adults and rare yet serious diseases presents a diagnostic challenge. Recognizing red flags can facilitate the diagnosis in such cases. Firstly, this patient reported worsening, well-localized pain that progressed over months, involving multiple bony areas of the chest wall, unlikely to be related to heavy lifting. Secondly, his symptoms persisted despite analgesic treatment, necessitating multiple visits to urgent care. Notably, his significant family history of prostate cancer provided a crucial clinical clue, raising suspicion of a more sinister etiology for his chest pain. As initially thought, EOPC generally carried better outcomes and a lower mortality rate due to fewer comorbidities in younger patients who can tolerate aggressive treatment options. However, more recent data has shown that EOPC is more likely to present with metastatic disease, resulting in a lower survival rate. In contrast, LOPC typically develops metastasis at later stages, with slower disease progression, allowing many patients to achieve long-term survival even with metastasis. Therefore, EOPC generally leads to poorer survival outcomes compared to LOPC. This is supported by a literature review suggesting that younger patients with high-grade, locally advanced disease are three times more likely to succumb to the disease than older men with similar disease burdens [12]. The overall five-year relative survival rate in the United States for men diagnosed between ages 40 and 80 years was between 95% and 100%. However, it was 30% for those aged 15 to 24 years, 50% for those aged 20 to 29 years, and 80% for those aged 25 to 34 years [13]. Hence, physicians must exercise vigilance when treating young patients presenting with unusual disease symptoms, as early diagnosis can facilitate life-saving interventions.

## Conclusions

In recent years, the incidence of EOPC has risen, increasing the risk of more aggressive disease. Distant metastasis can lead to unusual manifestations of the disease, such as neurological, musculoskeletal, and dermatological symptoms and signs, which may appear distant from the primary cancer site and often precede genitourinary symptoms. Considering its unusual presentation and significant genetic predisposition, individuals at high risk due to a strong family history should be monitored through personalized screening plans.
